# Strengthening speech development through timely delivery of orthodontic care: a narrative review

**DOI:** 10.3389/froh.2026.1938356

**Published:** 2026-10-26

**Authors:** Saurabh RamBihariLal Shrivastava, Prateek Sudhakar Bobhate, Sonali Borkar, Prithvi Brahmanand Petkar, Adeeba Siddique, Punit Fulzele

**Affiliations:** 1 Department of Community Medicine, Datta Meghe Medical College, Adani Institute of Higher Education and Research, Nagpur, Maharashtra, India; 2 Dr. D. Y. Patil School of Allied Health Sciences, Dr. D. Y. Patil Vidyapeeth, Pune (Deemed to be University), Pimpri, Pune, Maharashtra, India; 3 Additional Professor, Department of Community Medicine, All India Institute of Medical Sciences, Vijaypur, Jammu, India; 4 Department of Dentistry, Datta Meghe Medical College, Adani Institute of Higher Education and Research, Wanadongri, Nagpur, Maharashtra, India; 5 Department of Pediatric and Preventive Dentistry, Sharad Pawar Dental College, Adani Institute of Higher Education and Research, Wardha, Maharashtra, India

**Keywords:** children, dental, dental malalignment, malocclusion, orthodontics, schools, speech, speech-language pathologists

## Abstract

Globally, orthodontic treatment among children and adolescents has reported a remarkable rise, predominantly due to increased awareness about dental esthetics, improvement in dental functions, and most importantly, the benefits attributed to early intervention. This article is based on a narrative, evidence-informed synthesis of published literature on improving speech development through timely delivery of orthodontic care. A PubMed search was conducted to identify studies on speech development and orthodontic care in children using the keywords ’speech disorders’, “malocclusion”, “orthodontic”, and “children” in the title field. Ultimately, 34 articles were included based on their relevance to the review objectives and analyzed. During childhood, speech development and dental changes occur simultaneously, and early dental interventions become crucial in supporting optimal speech development and vice versa. Acknowledging the magnitude of the problem, the long-term impacts on speech development due to misalignment, and the availability of cost-effective orthodontic interventions, there is an indispensable need to strengthen this domain to bring about an improvement in articulation, resonance, and speech clarity. In conclusion, malocclusion and other dentofacial abnormalities may be associated with alterations in speech production; however, the percentage of speech disorders directly attributable to malocclusion remains uncertain. Moreover, speech development is multifactorial and should not be attributed solely to dental alignment. This essentially requires the timely implementation of combined orthodontic and speech-language pathologists’ services to improve both functional and esthetic outcomes.

## 1 Introduction

Globally, orthodontic treatment among children and adolescents has reported a remarkable rise, predominantly due to increased awareness about dental esthetics, improvement in dental functions, and most importantly, the benefits attributed to early intervention (1, 2). The findings of a cross-sectional study done among children and adolescents in Germany revealed that almost 26% and 21% of girls and boys, respectively, received orthodontic treatment, and there was a significant increase in orthodontic treatment across study participants from all age groups (1). Another study in Southern Italy revealed that almost 20% of pediatric age groups expressed the need for orthodontic treatment (2). However, we must not forget that multiple factors determine access to orthodontic treatment, including socioeconomic status, and there arises the need to implement public health interventions to promote optimal oral health (1, 2).

Speech development and dentofacial attributes (like anterior open bite, posterior crossbite, increased overjet, and altered tongue posture) are closely interrelated during childhood, establishing enough grounds for an important interface between orthodontic and speech-language care (3–5). A systematic review suggested association between specific malocclusions and articulation disorders, nevertheless, the underlying relationships are not completely deciphered (6). Further, as speech development is multifactorial, and influenced by hearing, neuromotor development, oral-motor function, craniofacial anomalies, etc., we must examine orthodontic and speech-related factors together to ascertain the exact cause and plan an appropriate treatment (7–9). The objectives of the current review are to enumerate the role of dental structures in speech development, identify the scope of orthodontic treatment modalities and their impact on speech outcomes, and propose cost-effective orthodontic interventions that can bring about an improvement in articulation, resonance, and speech clarity.

## 2 Methodology

This article is based on a narrative, evidence-informed synthesis of published literature on improving speech development through timely delivery of orthodontic care. A PubMed search was conducted to identify studies on speech development and orthodontic care in children. Relevant articles published between 2017 and 2026 were included. Initially, 126 research articles were identified using the keywords 'speech disorders', “malocclusion”, “orthodontic”, and “children” in the title field (namely speech [ti] AND malocclusion [ti]; oral [ti] AND malocclusion [ti]; speech [ti] AND orthodontic [ti]; speech [ti] AND children [ti]; orthodontic [ti] AND children [ti]; and speech [ti]). Only articles published in English were included. Of the 126 records screened, 86 were excluded because they did not meet the objectives of this review, and six were excluded because the full text was unavailable. Ultimately, 34 articles were included based on their relevance to the review objectives and analyzed (Figure 1).

**Figure 1:**
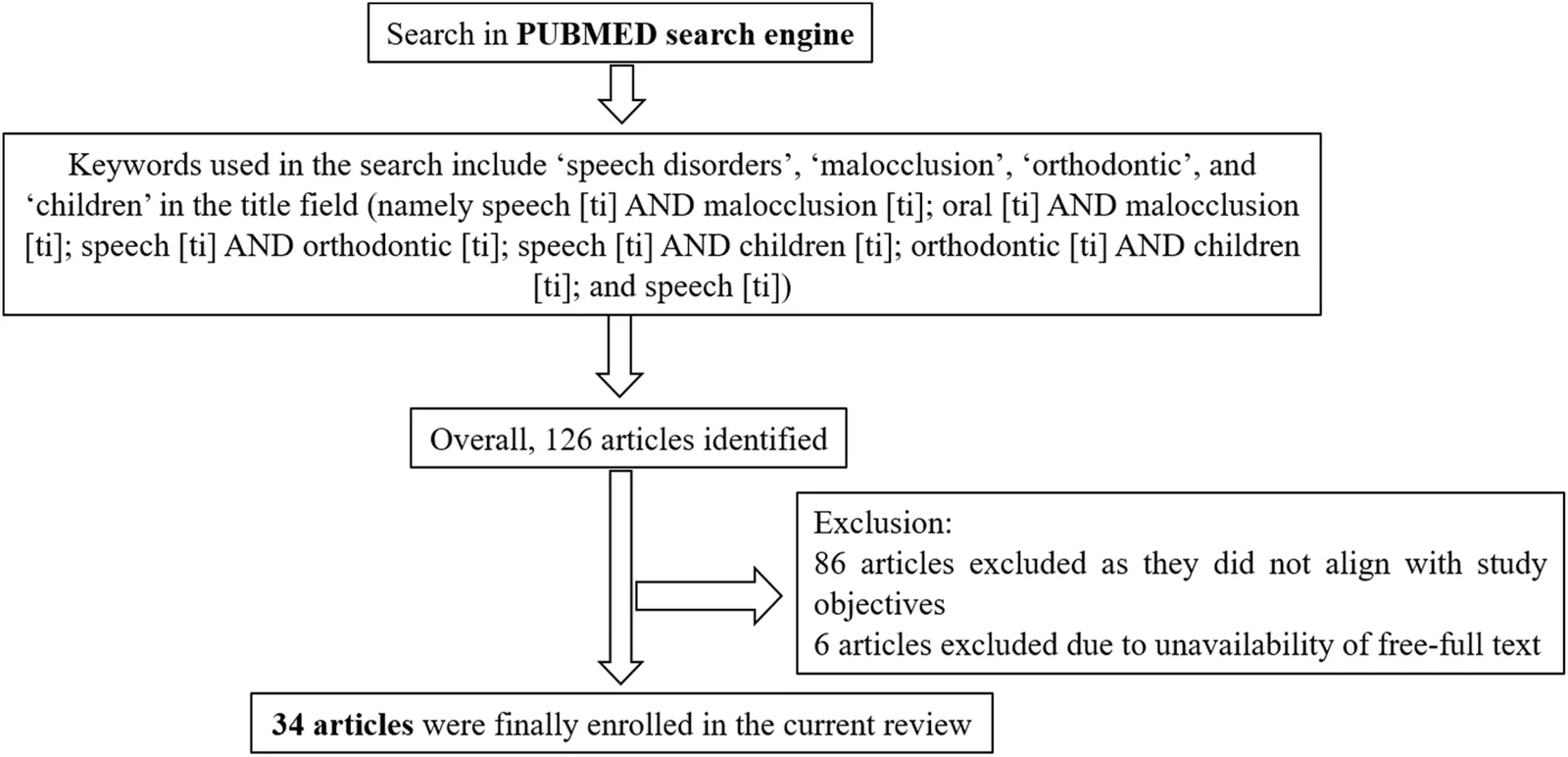
Flowchart for search of research articles.

## 3 Speech development and dental structures

Speech is fundamental for sharing thoughts, feelings, and needs, and is closely linked with cognitive growth through language development (10). At the same time, speech enables children to engage with peers, participate in social activities, engage in classroom discussions, and accomplish literacy milestones (11). Further, communicating effectively boosts self-esteem and confidence, facilitating participation in both academic and social settings (12). Dental structures are crucial in speech development in multiple ways, beginning with the positioning of teeth act as contact points for the tongue and lips, impacting the clarity of sounds (13). In other words, malocclusion (like overbites, crossbites, open bites, etc.) interferes with the placement of the tongue and airflow, affecting sound production (14). Further, crowded teeth or a narrow dental arch can restrict tongue motion, resulting in improper articulation and sound alterations (13) Likewise, a well-formed hard palate is essential for proper articulation and resonance, while jaw size determines the relationship between the tongue, teeth, and lips, influencing speech sound (13–15). Moreover, early loss of primary teeth or habits (such as thumb-sucking or prolonged use of a pacifier) can affect speech clarity and development (16).

The available literature does support a positive association between malocclusion and alterations in speech production, nevertheless this evidence does not indicate causality (Table 1) (3–5, 14). In a cross-sectional study done in Brazil among 547 mixed-dentition schoolchildren, it was reported that participants with anterior open bite were 2.3 times more likely to exhibit an altered tongue position [95% confidence interval (CI) 1.59–3.49], while posterior crossbite was also associated with a greater prevalence of speech distortion (prevalence of 1.77; 95% CI 1.09–2.88) (3). These findings suggest that specific occlusal characteristics, and not just malocclusion as a single entity, may influence tongue posture and speech production (3). In another cross-sectional study conducted among 290 children aged 4–7 years, a statistically significant association was identified between malocclusions (including anterior open bite, edge-to-edge bite, increased overjet, anterior crossbite, etc.) and phonetic changes (*p* < 0.05) (4).

**Table 1:** Recent empirical evidence concerning malocclusion, orthodontic treatment, and speech outcomes.

Studies	Country/Location	Design/Population	Main findings	Interpretation
Assaf et al., 2021 (3)	Santa Maria, Brazil	Cross-sectional epidemiological study; 547 children aged 7–13 years	Anterior open bite associated with altered tongue position (PR 2.36; 95% CI 1.59–3.49); posterior crossbite associated with speech distortion (PR 1.77; 95% CI 1.09–2.88)	Supports phenotype-specific associations between malocclusion, tongue position, and speech distortion; does not establish causality
Melo et al., 2021 (21)	Londrina, Brazil	Parallel randomized clinical trial; 40 patients with Class I malocclusion and moderate crowding	Clear aligners produced greater early speech alterations than fixed appliances (*p* < 0.001); differences disappeared by day 30	Suggests that appliance-related speech changes are predominantly transient and may resolve with adaptation
Amr-Rey et al., 2022 (4)	Valencia, Spain	Cross-sectional study; 290 children aged 4–7 years	Malocclusion associated with phonetic alterations (*p* = 0.008); sigmatism and rhotacism frequently observed	Supports an association between specific malocclusions and phonetic alterations during early childhood but does not establish causation
Mogren et al., 2022 (5)	Sweden	Cross-sectional clinical study; including 61 with speech sound disorders and 44 with typical speech development	Malocclusion in 61% of children with speech-disorder group vs. 29% with typical speech group	Demonstrates greater co-occurrence of malocclusion among children with speech sound disorders and supports multiprofessional assessment
Keyser et al., 2022 (23)	Chapel Hill, North Carolina, United States of America	Clinical speech/ acoustic investigation in skeletal anterior open bite	Speech abnormalities were substantially more frequent in patients with dentofacial deformity; perceptual and spectral abnormalities were investigated	Provides objective perceptual and acoustic evidence of speech differences associated with skeletal anterior open bite; does not quantify orthodontic treatment-related improvement
Tashkandi et al., 2025 (14)	Riyadh, Saudi Arabia	Cross-sectional study; 100 participants	27 participants demonstrated improper articulation; Peer assessment rating scores were significantly associated with selected sounds (*p* = 0.004, 0.017 and 0.01)	Supports an association between malocclusion severity and selected articulation abnormalities; the adult sample limits direct generalization to children

Evidence obtained from a study involving 105 Swedish children reported that malocclusion was present in 61% of the study participants with speech sound disorders and motor speech involvement compared with 29% of subjects with typical speech development (15). Another study aimed to evaluate severity of malocclusion using the peer assessment rating index among 100 participants demonstrated a statistically significant association between increasing malocclusion scores and improper articulation of selective speech sounds (14). These study findings cumulatively support an association between dentofacial morphology and speech production. However, as most available studies are observational and cross-section in original, these findings should not be interpreted as if malocclusion independently resulted in speech disorders. There is a need to conduct longitudinal studies, incorporating standardized orthodontic and speech-language assessments to establish temporality and causal associations.

However, it is essential to acknowledge that speech development is multifactorial, and dental alignment represents only one of several anatomical, sensory, neuromotor, developmental, and environmental factors that may influence speech production. A 2022 systematic review included 13 studies assessing speech, language, and literacy outcomes in children with mild-to-moderate hearing loss reported poor performance than hearing peers in multiple speech and language domains, although the review emphasized substantial heterogeneity (7). A recent clinical cohort study evaluate 198 children with speech sound disorders and 77 age-matched controls and examined 31 medical, developmental, oral-behavioural and hereditary risk factors (8). This study reported that children with speech sound disorders may have multiple coexisting developmental and oral-motor characteristics, justifying the need for comprehensive assessment (8). In addition, other factors, including developmental language disorders, oral-motor dysfunction, craniofacial anomalies, neurological conditions, respiratory and oral habits, linguistic and environmental context of the child, may also contribute to speech outcomes (Table 2) (7–9). This calls for the need to potentially consider these confounders during assessment of such children and should preferably involve a multidisciplinary approach.

**Table 2:** Major non-orthodontic factors that may influence speech development.

Factors	Potential relationship with speech	Evidence/Clinical consideration
Hearing impairment	Reduced auditory input may affect speech perception, production and language development	Children with mild-to-moderate hearing loss have demonstrated poorer performance in some speech and language domains, although findings are heterogeneous
Motor speech/developmental delay	Neuromotor limitations may affect speech-motor planning, articulation and intelligibility	Children with speech sound disorders may have coexisting motor speech and oral-motor developmental difficulties
Developmental language disorder	Language impairment may coexist with or contribute to speech difficulties	Speech assessment should distinguish speech sound problems from broader language impairment
Ankyloglossia (tongue-tie)	Restricted tongue mobility may potentially affect selected articulatory movements	Evidence linking ankyloglossia directly to speech remains inconsistent and generally low quality
Craniofacial anomalies	Structural differences may affect articulation, resonance and oral-motor function	Particularly relevant in children with cleft lip/palate and other craniofacial conditions
Oral habits/respiratory pattern	Persistent oral habits and altered oral posture may affect orofacial function and articulation	Should be assessed alongside dental occlusion
Neurological/developmental disorders	May affect motor planning, muscle control, language and speech production	Requires multidisciplinary developmental assessment

## 4 Orthodontic treatment modalities and speech outcomes

During childhood, speech development and dental changes occur simultaneously, and early dental interventions become crucial in supporting optimal speech development and vice versa (17). To improve speech and assist in speech development, multiple common orthodontic interventions can be prescribed depending on the indications and feasibility (18). Fixed appliances (braces) can be used to treat crowding, spacing, and malocclusion, while functional appliances are used to correct jaw discrepancies (18, 19). Clear aligners can also be employed to correct dental malalignment, and it is preferred for esthetic reasons, easier oral hygiene, and dental flexibility (20). The available empirical evidence does indicate that orthodontic appliances themselves can alter speech transiently during the adaptation period (Table 1) (21). In fact, in a parallel, randomized clinical trial wherein orthodontic aligners were compared with conventional fixed appliances, a significant change in speech production was reported with orthodontic aligners immediately upon their placement and at 3 days (*p* < 0.001) (21).

However, no such objective differences were reported between the two groups after 30 days (*p* = 0.487) or 180 days, and this indicated that appliance-related speech alterations are more predominant during the early adaptation period rather than causing persistent impairment (21). Nevertheless, evidence relating to other orthodontic interventions remains heterogeneous (22). A systematic review of rapid maxillary expansion involving seven interventional studies including almost 200 patients, reported changes in acoustic speech parameters (like vowel fundamental frequency and fricative formant frequencies) during treatment (22). On the other hand, two studies reported deterioration, while some studies reported temporary changes during active treatment followed by improvement after removal of the appliances (22). In other words, these findings should not be interpreted that orthodontic treatment alone improves a pre-existing developmental speech disorder.

Orthodontic appliances may cause transient speech alterations, especially during the initial adaptation period after appliance placement (23). Changes in articulation of sibilant sounds and temporary lisping have been reported with clear aligners and other orthodontic appliances (21). At present, there is inadequate evidence to mention a specific percentage of speech improvement or prevention of speech deterioration that can be attributed to orthodontic treatment alone in children with malocclusion. It is anticipated that future longitudinal studies report standardized pre-treatment and post-treatment speech outcomes, the percentage of subjects demonstrating clinically meaningful improvement, the proportion with persistent or worsened speech abnormalities, and the share of concurrent speech-language therapy in overall improvement. The availability of such data can aid in differentiating the effects of orthodontic correction from natural developmental changes and from the effects of speech-language interventions.

## 5 Proposed interventions

Acknowledging the magnitude of the problem, the long-term impacts on speech development due to misalignment, and the availability of cost-effective orthodontic interventions, there is an indispensable need to strengthen this domain to bring about an improvement in articulation, resonance, and speech clarity (7–9, 13–18, 24–34). This has to begin with running awareness campaigns to educate parents on the importance of oral health, its connection with speech development, and the necessity to detect and treat at the earliest (24, 25). The educational materials for these awareness activities must be created in multiple languages to reach culturally diverse populations (25). There is an immense need to integrate dental health education and preventive measures into preschool and daycare curriculum, as this will inculcate oral health promotion in the early childhood phase (26). In continuation, educational programs can be implemented in schools to encourage healthy oral habits (viz. discourage thumb-sucking, use of pacifier, and mouth breathing), which will reduce the possibility of malocclusion and associated speech difficulties (16, 26).

The need of the hour is to implement regular dental and speech assessments in school settings to identify children with malocclusions and speech delays, and refer them to specialists (27). Children with known risk factors (like cleft palate, etc.) should be targeted first for early orthodontic and speech assessments to avoid long-term consequences (28). Space maintainers can aid in maintaining dental arch relationships, and this space preservation may reduce one potential dentofacial factor that could subsequently contribute to altered oral function or speech (18, 29). There is a definite need to encourage collaboration between orthodontists, speech-language pathologists, pediatricians, and educators to address oral and speech health holistically (15–17) In this regard, clinics that offer a combination of both orthodontic and speech-language pathology services can be established, and they can provide comprehensive care for children with overlapping requirements (13, 14, 17). Although early orthodontic assessment may identify dentofacial risk factors before functional problems become established, insufficient evidence is available which suggests that orthodontic treatment prevents future speech deterioration. In other words, preventive benefits should be acknowledged as a clinical rationale for early assessment than effect of treatment, unless supported by longitudinal comparative data.

From the health sector perspective, orthodontists, speech-language pathologists, and primary care providers must be given specialized training to recognize and manage dental-speech interactions efficiently and effectively (13, 14). Before attributing a speech difficulty to malocclusion, a comprehensive assessment must rule out potential non-orthodontic contributing factors and confounders, as such approach can minimize the risk of attributing multifactorial speech difficulties solely to dental alignment (7–9). In addition, community health workers can also be trained to provide basic oral health education, screen children in underserved areas, and refer children for immediate specialist attention (30). To enhance access and equity, government-funded or subsidized services or insurance coverage can be offered to people from low-socioeconomic families for both orthodontic treatment and speech therapy (31). Also, the option of telehealth or mobile apps can be used to deliver educational content, reminders for checkups, and virtual consultations for dental and speech concerns (32). There is a significant need to conduct research work, and based on the findings obtained, customized strategies for prevention, early detection, and management of malocclusions and speech disorders can be framed (17, 18). In this regard, registries and surveillance systems can be established to track the prevalence of orthodontic issues and speech disorders, and the impact of interventions (33, 34).

The reported evidence must be interpreted cautiously, as most of the cited literature examining malocclusion and speech are from cross-sectional observational studies, which can either demonstrate coexistence or statistical association but cannot determine the direction of causality. Moreover, speech production is multifactorial and may also be influenced by age, language, neuromotor development, oral habits, respiratory pattern, craniofacial morphology, and previous speech-language intervention. Also, owing to the heterogeneity reported in malocclusion classification, speech-assessment methods, languages studied, and outcome measures, a direct comparison across studies cannot be made. There is an urgent need to conduct longitudinal and controlled interventional studies using standardized orthodontic and speech-language outcomes to draw meaningful inferences and establish causality.

## 6 Conclusion

In conclusion, malocclusion and other dentofacial abnormalities may be associated with alterations in speech production; however, the percentage of speech disorders directly attributable to malocclusion remains uncertain. Moreover, speech development is multifactorial and should not be attributed solely to dental alignment. This essentially requires the timely implementation of combined orthodontic and speech-language pathologists' services to improve both functional and esthetic outcomes.
